# Designing provider-focused implementation trials with purpose and intent: introducing the PRECIS-2-PS tool

**DOI:** 10.1186/s13012-020-01075-y

**Published:** 2021-01-07

**Authors:** Wynne E. Norton, Kirsty Loudon, David A. Chambers, Merrick Zwarenstein

**Affiliations:** 1 Division of Cancer Control and Population Sciences, National Cancer Institute, 9609 Medical Center Drive, #3E424, Bethesda, MD 20850 USA; 2 Independent Contractor, Edinburgh, Scotland; 3 Centre for Studies in Family Medicine, Department of Family Medicine, Western University, London, Ontario Canada

**Keywords:** Pragmatic trials, Explanatory trials, Implementation science, Randomized controlled trials, PRECIS, Implementation strategies, Multi-level trials, Healthcare delivery research

## Abstract

**Background:**

First articulated by Schwartz and Lellouch (1967), randomized controlled trials (RCTs) can be conceptualized along a continuum from more explanatory to more pragmatic. The purpose and intent of the former is to test interventions under ideal contexts, and the purpose and intent of the latter is to test interventions in real-world contexts. The *PR*agmatic *E*xplanatory *C*ontinuum *I*ndicator *S*ummary-2 (PRECIS-2) is a validated tool that helps researchers make decisions about the elements of the trial to match the overall purpose and intent of the trial along the continuum. The PRECIS-2 tool has guided the design of hundreds of RCTs. However, a few aspects of the tool would benefit from greater clarity, including its application to provider-focused implementation trials rather than patient-focused intervention trials.

**Main text:**

We describe the newly developed PRECIS-2-Provider Strategies (PRECIS-2-PS) tool, an extension of the PRECIS-2 tool, which has been adapted for trials testing provider-focused strategies. We elaborate on nine domains that can make a provider-focused trial more explanatory or more pragmatic, including eligibility, recruitment, setting, implementation resources, flexibility of provider strategies, flexibility of intervention, data collection, primary outcome, and primary analysis. We detail the complementary roles that researchers and stakeholders play in the trial design phase, with implications for generalizability of trial results to the contexts in which they are intended to be applied.

**Conclusions:**

The PRECIS-2-PS tool is designed to help research and practice teams plan for provider-focused trials that reflect the overall intent and purpose of the trial. The tool has potential to help advance the science of provider-focused strategies across a range of trials, with the ultimate goal of facilitating the adoption, integration, and sustainability of provider-focused strategies outside the context of trials.

**Supplementary Information:**

The online version contains supplementary material available at 10.1186/s13012-020-01075-y.

Contributions to the literature

- Tools can help plan for randomized controlled trials to match the overall intent and purpose of the trial.
- The PRECIS-2 tool helps plan for trials along a continuum from ideal (explanatory) to real-world (pragmatic) settings. The PRECIS-2 tool focuses on trials testing patient-level interventions but not provider-focused strategies.
- We extend the PRECIS-2 tool to trials testing provider-focused strategies and describe how to interpret the nine domains of the PRECIS-2- Provider Strategies (PRECIS-2-PS) tool.
- We describe the collaborative role that researchers and stakeholders play in using the tool to match the intent and purpose of the trial to the design of the trial.

## Background

Randomized controlled trials (RCTs), including individual-, cluster-, and stepped-wedge, are considered the “gold standard” study design for identifying effective health-related innovations [1]. Compared to other types of study designs (e.g., quasi-experimental), RCTs are best equipped to minimize the multitude of threats to internal validity that can compromise the integrity and interpretation of trial results [2]. However, RCTs are often criticized for having poor external validity—that is, trial results do not accurately reflect the circumstances to which the results should or could be applied once the study is completed. The potential for trials to impact patient outcomes, care delivery, and population health is limited if trial results are neither generalizable nor applicable to the contexts in which they are intended to apply.

In recognition of this criticism, methodologists have considered ways in which RCTs can be designed to better balance both internal and external validity. First introduced by Schwartz and Lellouch (1967), one way of conceptualizing trials with more or less emphasis on external validity is along a multi-axial continuum from more explanatory to more pragmatic [3]. On one end of the continuum, *explanatory* trials are those that emphasize internal validity. Explanatory trials seek to understand if an intervention is effective under *ideal* contexts, which are often characterized as highly resourced, tightly controlled, and conducted in somewhat artificial settings. These trials are mainly concerned with understanding and testing hypotheses on the existence of particular mechanisms of action for a given innovation. On the other end of the continuum are *pragmatic* trials. These trials emphasize a balance between internal and external validity. Pragmatic trials seek to understand if innovations work in *real-world* contexts that closely reflect the settings in which the innovation is intended to be used [4].

Interest in pragmatic RCTs (pRCTs) has increased substantially in recent years as researchers, practitioners, and funders continue to recognize the need for innovations that are both effective and generalizable beyond a single trial. Many opportunities now exist to support the conduct of pRCTs, including educational courses (e.g., massive open online course, *Pragmatic Randomized Controlled Trials in Health Care*, hosted by edX [5]), trainings and research networks (e.g., National Institutes of Health [NIH] Health Care Systems Research Collaboratory [6]), online resources (e.g., interactive eBook, *Pragmatic Trials: A Workshop Handbook* [7];), and funding opportunity announcements [8, 9]. Tools for helping research teams plan for trials along the explanatory-pragmatic continuum to match the overall intent and purpose of the trial are also available. One such tool is the *PR*agmatic *E*xplanatory *C*ontinuum *I*ndicator *S*ummary-2 (PRECIS-2 [10]).

The PRECIS tool was first developed by Thorpe and colleagues (2009) and revised by Loudon and colleagues (2015) through a collaborative, iterative process involving 80 international trialists [10, 11]. Briefly, the PRECIS-2 tool operationalizes elements or characteristics of trials that make them more or less pragmatic; in doing so, it encourages trialists to make purposeful decisions about the trial design to match the intent and purpose of the trial along the explanatory-pragmatic continuum. PRECIS-2 identifies nine domains of a trial that can make it more explanatory or more pragmatic: eligibility, recruitment, setting, organization, flexibility (delivery), flexibility (adherence), follow-up, primary outcome, and primary analysis.

The PRECIS-2 tool is intended to be used by the research team when planning for a trial. Through interactive, team-based discussions, each of the nine domains of the tool are scored on a five-point scale, with a score of one reflecting characteristics of that domain as very explanatory and a score of five reflecting characteristics of that domain as very pragmatic. Scores for each trial domain are represented on a wheel, where trials that are more explanatory have scores toward the center of the wheel, and trials that are more pragmatic have scores toward the periphery of the wheel. Scores for each domain can vary across the explanatory-pragmatic continuum, such that a score for one domain in one trial may be very pragmatic and a score for another domain within the same trial may be very explanatory. Rarely are all nine domains in a single trial scored as a one (very explanatory) or scored as a five (very pragmatic). Scores may also fluctuate throughout the duration of the trial, as planned or unplanned changes to specific domains may be necessary (e.g., recruitment, eligibility). As there are no objectively preferred individual or collective domain scores for a trial, each domain should match the intent and purpose of that particular trial.

The PRECIS-2 tool has been used to help design over 500 RCTs (personal communication, K. Loudon, August 15, 2020), with hundreds more retrospective assessments reported in published papers [12, 13]. It has demonstrated good interrater reliability and modest discriminant validity [14] and is often a major component of trainings and workshops for both research teams [5, 6] and funding agency staff [15]. With increased use and application of the PRECIS-2 tool to a wide range of trials, some users have identified a few aspects of the tool that would benefit from greater clarity and guidance.

One common source of confusion encountered by users of the PRECIS-2 tool is how to apply and interpret all nine domains to trials where participants are healthcare professionals (rather than patients) and trials are testing provider-focused strategies (rather than health interventions [16, 17]). While the eligibility and recruitment domains are similarly interpreted for trials involving patients or providers, it is less clear how to differentiate and operationalize all domains for trials testing health interventions that target patients compared to trials testing strategies that target healthcare professionals. Although the PRECIS-2 toolkit [10] states that “participants may be patients and/or healthcare professionals, interventions may target patients (e.g., medication) or healthcare professionals (e.g., continuing education),” it stops short of specifying how to conceptualize and interpret all domains for trials with healthcare professionals and provider-focused strategies. As trials designed to test provider-focused strategies vary from those that test patient-level interventions, the PRECIS-2 tool should vary, as well.

To clarify this issue, we propose an extension and adaptation of the PRECIS-2 tool tailored to trials that test strategies to change providers’ behavior. Such trials are quite common in implementation science, healthcare delivery research, and quality improvement research, where the overall goal is often to increase the adoption of evidence-based health interventions, de-implement ineffective interventions, and/or improve healthcare delivery and service provision. Consistent with the literature, we consider provider-focused strategies as those that target providers’ behavior; examples include audit and feedback, continuing education, and external coaching, among others [18].

The PRECIS-2-Provider Strategies (PS) tool was developed through a multi-step process involving experts from the original PRECIS tool (2009; MZ), revised PRECIS-2 tool (2015; KL, MZ), and implementation scientists (WEN, DAC). A series of 2-h discussions were held to deliberate the proposed domains, descriptions, and scores of PRECIS-2-PS. The PRECIS-2 worksheet and domain examples were used as points-of-comparison to contrast domains for trials where participants are patients and the target of health interventions *vs*. trials where participants are providers and the target of strategies. We leveraged our content expertise and trial experience to generate examples of a score of 1 (more explanatory) and a score of 5 (more pragmatic) for all nine domains. While formal reliability and validity testing was considered beyond the scope of the preliminary development of the tool, we nonetheless applied the PRECIS-2-PS tool to a small sample of four diverse trial protocols for pilot testing. Three of us independently read each trial protocol, scored each domain, and highlighted relevant text that informed the domain score. Scores and selected text were compared across coders; discrepancies were discussed until consensus was reached, and refinements to the tool were made accordingly (e.g., examples of domain scores). The implementation resources (#4) and data collection (#7) domains generated the most discussion. As the conceptualization of these two domains deviates the most from PRECIS-2, additional discussion of scores and refinement of PRECIS-2-PS was not unexpected. The remaining seven domains (e.g., eligibility, recruitment) generated fewer discrepancies and discussions.

We describe the PRECIS-2-PS tool below. We define and operationalize each of the nine domains of the PRECIS-2-PS tool and provide examples of characteristics that make that domain more explanatory (score 1) or more pragmatic (score 5). We provide guidance for how to use the tool during the trial planning phase, including a comprehensive toolkit (see Additional file). Finally, we build on our experience using the PRECIS-2 tool in training workshops and trial consultations to emphasize emergent issues and apply them to the PRECIS-2-PS tool. These include the complementary role of different stakeholder groups for trial design and the importance of understanding and describing implementation-as-usual.

## Discussion

### PRECIS-2-PS domains

**Fig. 1:**
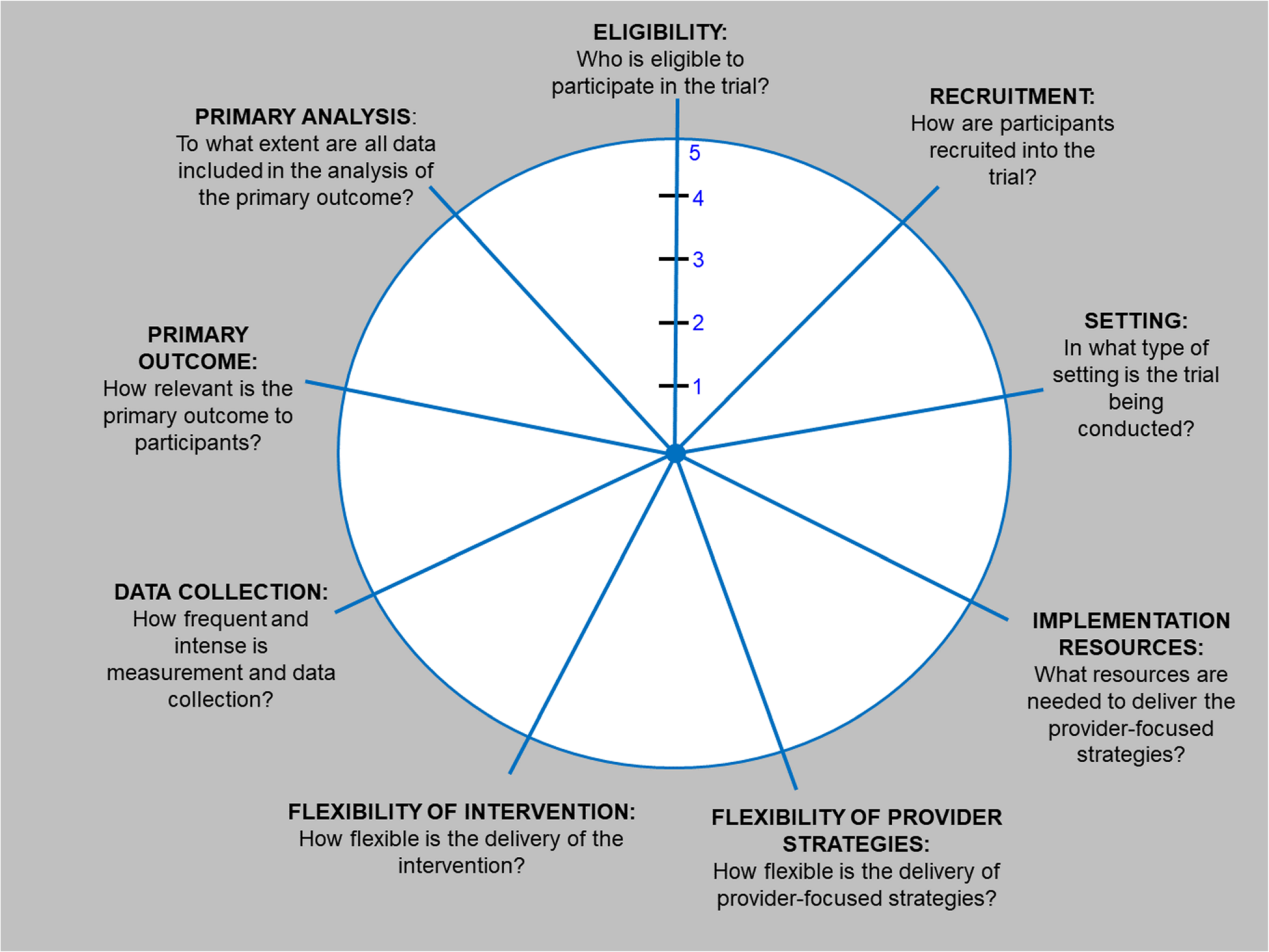
The PRagmatic-Explanatory Continuum Indicator Summary 2 Provider Strategies (PRECIS-2-PS) wheel

Consistent with the PRECIS-2 tool, we include a total of nine domains for PRECIS-2-PS. We changed the domain name, key question, and/or description for all domains to match the specific nature of provider-targeted trials. A comparison on domain names, key questions, and score examples between PRECIS-2 and PRECIS-2-PS can be found in Table 1. We used the same wheel-and-spoke visualization of PRECIS-2 for the PRECIS-2-PS tool. A blank version of the PRECIS-2-PS wheel is shown in Fig. 1.

**Table 1:** Comparison of PRECIS-2 and PRECIS-2-PS on domains, key questions, and scores

PRECIS-2	PRECIS-2-PS
**Eligibility** *To what extent are the patients in the trial similar to those who would receive this intervention if it was part of usual care?* Score 1 for a very explanatory approach with lots of exclusions for patients (e.g., those who don’t comply, respond to treatment, or are not at high-risk for primary outcome, are children or elderly), or uses many selection tests not used in usual care. Score 5 for very pragmatic criteria essentially identical to those in usual care.	**Eligibility** *To what extent are the healthcare professionals in the trial similar to those in usual care?* Score 1 for a very explanatory approach that only allows for a select, narrow, and non-representative sample of healthcare professionals. Score 5 for a very pragmatic approach with broad inclusion criteria and minimal exclusion criteria for healthcare professionals.
**Recruitment** *How much extra effort is made to recruit patients over and above what would be used in the usual care setting to engage with patients?* Score 1 for a very explanatory approach with targeted invitation letters, advertising in newspapers, radio plus incentives and other routes that would not be used in usual care. Score 5 for very pragmatic recruitment through usual appointments or clinic.	**Recruitment** *How much extra effort is made to recruit healthcare professionals into the trial compared to what is available to encourage their engagement in usual care settings?* Score 1 for a very explanatory approach with extensive approaches (e.g., personalized invitation letters, free CME credits, personalized outreach from physician opinion leaders, support or endorsement from professional societies) that would not otherwise be available or used in usual care. Score 5 for a very pragmatic approach with feasible approaches that leverage information channels (e.g., flyers posted in break rooms, emails to staff, word-of-mouth, lunch discussions/seminars) that are commonplace and available in usual care settings.
**Setting** *How different is the setting of the trial* (*or are the settings of the trial*) *from the usual care setting?* Score 1 for a very explanatory approach with only a single center, or only specialized trial or academic center. Score 5 for a very pragmatic choice using identical settings to usual care.	**Setting** *How different is the health care or public health setting* (*e.g., hospital, clinic, health department*) *in which the trial is conducted compared to usual care settings?* Score 1 for a very explanatory approach that includes sites in a limited geographic region, lack of diversity in organizational characteristics, few number of sites, and/or a single delivery setting or system. Score 5 for a very pragmatic approach that includes a representative sample of sites in terms of location, number, size, system, and organizational characteristics.
**Organization** *How different are the resources, provider expertise, and the organization of care delivery in the experimental arm of the trial and those available in usual care?* Score 1 for a very explanatory approach if the trial increases staff levels, gives additional training, requires more than usual experience or certification and increases resources. Score 5 for a very pragmatic choice that uses identical organization to usual care.	**Implementation resources** *How different are the resources needed to support the delivery of the provider-focused strategies from resources that are readily available in usual care?* Score 1 for a very explanatory approach if the trial provides more resources, time, and effort needed to deliver the provider-focused strategies (e.g., staff, financial incentives, training, equipment, consultation) than would otherwise be available in usual care. Score 5 for a very pragmatic approach if the trial uses resources, time, and effort to deliver the provider-focused strategies that are identical to those available in usual care.
**Flexibility (delivery)** *How different is the flexibility in how the intervention is delivered and the flexibility likely* (*or anticipated*) *in usual care?* Score 1 for a very explanatory approach if there is a strict protocol, monitoring, and measures to improve compliance, with specific advice on allowed co-intervention and complications. Score 5 for a very pragmatic choice with identical flexibility to usual care.	**Flexibility of provider-focused strategies** *How different is the flexibility in how provider-focused strategies are delivered in the trial and the flexibility in how provider-focused strategies are likely to be delivered in usual care?* Score 1 for a very explanatory approach if there is a strict protocol for how provider-focused strategies must be delivered (e.g., frequency, intensity, structure, process) and/or if adaptations to strategies are strongly discouraged. Score 5 for a very pragmatic approach that allows for flexibility and adaptation of provider-focused strategies.
**Flexibility (adherence)** *How different is the flexibility in how patients must adhere to the intervention* (*or are monitored and encouraged to adhere to the intervention*) *from the flexibility likely* (*or anticipated*) *in usual care?* Score 1 for a very explanatory approach that involves exclusion based on adherence, and measures to improve adherence if found wanting. In some trials (e.g., surgical trials), where patients are being operated on or ICU trials where patients are being given IV drug therapy, this domain is not applicable as there is no compliance issue after consent has been given, so this score should be left blank. Score 5 for a very pragmatic choice involving no more than usual encouragement to adhere to the intervention.	**Flexibility of intervention** *How different is the flexibility in how the intervention is delivered by healthcare providers to patients and the flexibility in how the intervention would be delivered in usual care?* Score 1 for a very explanatory approach that discourages intervention adaptation and encourages strict fidelity to the intervention. Score 5 for a very pragmatic choice that explicitly allows for or encourages adaptations to the intervention.
**Follow-up** *How different is the intensity of measurement and follow-up of patients in the trial and the likely follow-up in usual care (or from the typical follow-up in usual care)?* Score 1 for a very explanatory approach with more frequent, longer visits, unscheduled visits triggered by primary outcome event or intervening event, and more extensive data collection. Score 5 for a very pragmatic approach with no more than usual follow-up.	**Data collection** *How different is the frequency and intensity of measurement and data collection throughout the trial compared to what is considered routine in usual care?* Score 1 for a very explanatory approach that includes frequent, time-intensive, and extensive measurement and data collection. Score 5 for measurement and data collection that are routinely collected, readily available, or relatively easy to administer and obtain.
**Primary outcome** *To what extent is the trial’s primary outcome* (*directly*) *relevant to patients?* Score 1 for a very explanatory approach using a surrogate, physiological outcome, central adjudication, or use assessment expertise that is not available in usual care, or the outcome is measured at an earlier time than in usual care. Score 5 for a very pragmatic choice where the outcome is of obvious importance to patients.	**Primary outcome** *To what extent is the trial’s primary outcome important to healthcare professionals?* Score 1 for a very explanatory approach where the primary outcome variable is irrelevant or unimportant to healthcare professionals. Score 5 for a very pragmatic choice where the primary outcome is highly relevant and very important to healthcare professionals.
**Primary analysis** *To what extent are all data included in the analysis of the primary outcome?* Score 1 for a very explanatory analysis that excludes ineligible post-randomization patients, includes only completers or those following the treatment protocol. Score 5 for a very pragmatic approach using intention to treat with all available data.	**Primary analysis** *To what extent are all data included in the analysis of the primary outcome* Score 1 for a very explanatory approach where data analysis is limited to those participants who complete all measures and participate in all aspects of the trial (“on protocol”) and/or analysis that uses less robust and extensive imputation techniques to account for missing data. Score 5 for a very pragmatic approach using an intent-to-treat or modified intent-to-treat analytic approach with robust and limited imputation techniques.

*For both PRECIS-2 and PRECIS-2-PS, the comparison to usual care refers to the usual care settings in which the trial results would be applicable and ultimately used. Note that broad generalizability to all usual care settings, healthcare providers, and patients is neither assumed nor necessary for a trial to be pragmatic; rather, it is an assessment of the representativeness of the sample of care settings, healthcare providers, and patients in the trial to the full population of care settings, healthcare providers, and patients from which the sample is drawn, and to which the results are intended to be applied

#### Domain #1: Eligibility

The eligibility domain refers to characteristics of healthcare professionals who would qualify to participate in the trial. Healthcare professionals may include nurses, physicians, allied health professionals, specialists, patient navigators, social workers, community health workers, and other individuals who provide relevant services. Reflecting a very explanatory intent of the trial, a score of 1 for the eligibility domain would have extensive exclusion criteria. The sample of healthcare professionals eligible for and included in the trial would be a restricted subset of the full population of healthcare professionals to whom the findings of the trial would apply outside the context of a trial. Reflecting a very pragmatic intent of the trial, a score of 5 for this domain would have minimal exclusion criteria. The sample of healthcare professionals eligible for and included in the trial would be a representative subset of the full population of healthcare professionals to whom the findings of the trial would be expected to apply outside the context of a trial.

#### Domain #2: Recruitment

The recruitment domain refers to approaches used to recruit and enroll eligible healthcare professionals into the trial. The recruitment domain assesses to what extent additional time, effort, and resources are used to recruit participants into the trial compared to approaches that would be readily available in similar settings. Reflecting a very explanatory intent of the trial, a score of 1 would include extensive approaches in terms of additional time, effort, resources, and personnel for recruiting providers into the trial. Examples might include personalized invitation letters, free continuing medical education credits, monetary incentives, or other approaches that are uncommon or unavailable in routine care settings. Reflecting a very pragmatic intent of the trial, a score of 5 for this domain would include relatively few approaches. These approaches would be feasible in routine care settings, such as announcements at staff meetings, word-of-mouth, or flyers posted in break rooms.

#### Domain #3: Setting

The setting domain refers to characteristics of the setting (namely, organizations) in which the trial is conducted. Examples of organizations include hospitals, clinics, health centers, health departments, and community-based organizations, among others. Organizational characteristics may include location, size, resources, payment structures, culture, climate, and performance metrics. This domain assesses to what extent the organizations in the trial reflect organizations to which the results would apply; that is, are organizations included in the study a representative sample of organizations to which the results would apply, or are they unique in some way that makes them a poor representation of organizations to which the results are intended to apply?

Reflecting a very explanatory intent of the trial, a score of 1 for the setting domain would include a sample of organizations that are unusual or atypical in ways that make them a poor reflection of the full population of organizations to which the results are intended to be applied. Examples include organizations that are limited to a small geographic region, significantly under or over resourced, exceptionally high or low performers on quality metrics, or a single rarified delivery system. Reflecting a very pragmatic intent of the trial, a score of 5 for this domain would include organizations in the trial that closely represent the total population of organizations to which the trial is intended to be applied. Examples include organizations that are dispersed across a large geographic region, located in rural, urban, and peri-urban settings, and average performance on quality metrics.

#### Domain #4: Implementation resources

The implementation resources domain refers to the time, effort, and personnel needed to support the delivery of provider-focused strategies. This domain compares the intensity of strategies tested in the trial relative to what is or would likely be available to deliver those strategies outside the context of the trial. A very explanatory intent of the trial (score of 1) would reflect resources to support the delivery of strategies above and beyond what would be reasonable or available in similar care settings. This would include the use of strategies that are time-intensive, costly, extensive, and frequent, such as one-on-one in-person coaching sessions, and expensive expert-led external facilitation. A very pragmatic intent of the trial (score of 5) would use resources to deliver provider-focused strategies that are available or accessible in these settings, such as quality improvement teams and collaboratives, educational sessions, skill-building seminars or workshops, or electronic health record systems.

#### Domain #5: Flexibility of provider-focused strategies

The flexibility of delivery of provider-focused strategies domain refers to the flexibility in how, when, and by whom the strategies are delivered to providers within the trial. This domain reflects the extent to which individuals who deliver the strategies, and the detailed aspects of delivery of the strategies themselves, are pre-specified (e.g., training, experience, credentials), the format in which they can be delivered (e.g., in-person, online), the frequency and sequence in which they can be delivered, and the flexibility in selection, use, and adaptation of strategies. Reflecting a very explanatory trial, a score of 1 would include trials with strict protocols and structures that do not allow for or encourage adaptations to strategies, regardless of context. Reflecting a very pragmatic trial, a score of 5 would include trials with flexible guides, suggestions, and manuals for delivering provider-focused strategies that allow for or even encourage the adaptation of strategies to meet the context of the organization and the needs of healthcare professionals.

#### Domain #6: Flexibility of intervention

The flexibility of intervention domain refers to the degree to which healthcare professionals are able to adapt their use of the patient-focused intervention to their situation relative to the likely flexibility that would be available during usual care. A score of 1 on the flexibility of intervention domain, reflecting a very explanatory approach, would include strict protocols or structures in place to discourage or limit the extent to which the patient-focused intervention could be adapted by the provider. A score of 5 on the flexibility of intervention domain, reflecting a very pragmatic approach, would include explicit suggestions or encouragement for healthcare professionals to adapt the patient-focused intervention to the context.

#### Domain #7: Data collection

The data collection domain is a function of both the frequency and intensity of data collected throughout the duration of the trial from baseline through follow-up. This domain includes how often data are collected from participants as well as how extensive, intrusive, or time-consuming it is for participants to provide data compared to what would be considered routine within similar care settings. In a very explanatory trial (a score of 1), data would be collected quite often and require extensive time and effort on behalf of participants to complete. Very explanatory trials would also likely include (or be limited to) original data collection, including quantitative and/or qualitative data. In a very pragmatic trial (a score of 5), data would be collected less often and require little effort and time for completion. Very pragmatic trials would rely heavily on secondary data collection—that is, data that are readily available within routine care (e.g., electronic health records), with minimal or no original data collection.

#### Domain #8: Primary outcome

This domain is the extent to which the primary outcome is of interest and importance to healthcare professionals. Primary outcomes may include direct provider-level outcomes or indirect patient-level outcomes. Reflecting a very explanatory approach, a score of 1 on this domain would include trials where the primary outcome is some type of process or proxy variable, where the overall intent is to better explain or understand a mechanism or mediating variable that might predict the degree to which the provider-focused strategy is effective. Outcomes that would reflect a very explanatory trial include providers’ knowledge about an intervention, perceptions of leadership, or patients’ completeness of laboratory testing (where these tests may not be part of routine care). Reflecting a very pragmatic approach, a score of 5 on this domain would include trials where the primary outcome is of obvious interest and importance to healthcare professionals, such as their quality of care or job satisfaction, or their patients’ health status or quality of life.

#### Domain #9: Primary analysis

Primary analysis refers to the extent to which all data are used to assess the primary outcome of the trial. A trial that is more explanatory (a score of 1) would use an on-protocol approach to test the main study hypothesis, where only data from healthcare professionals who consented, received the provider-focused strategy (for experimental condition only), and completed all process and outcome measures of the study are included in the primary analysis. A more explanatory trial may also use less-robust data imputation techniques to account for attrition and missing data. Trials on the pragmatic end of the continuum (a score of 5) would use an intent-to-treat (ITT) or robust modified ITT analysis for comparing the trial arms on the primary outcome variable for all participants who consented into the trial [19–22].

### Enhancing the use of the PRECIS-2-PS tool

The PRECIS-2-PS tool is intended to be used during the trial planning phase to help team members consider structured, contextual elements of the trial that would match the overall intent and purpose of the trial. Building on our collective experience with PRECIS-2 and the published literature [23–25], we make explicit two major recommendations for enhancing the use of the tool that were not fully articulated in previous versions. The first recommendation is to involve stakeholders throughout the planning, execution, and interpretation of the trial. The second recommendation is to describe implementation-as-usual at the outset of the trial and document major changes that may occur as the trial unfolds. Together, we believe these approaches strengthen the ability of the tool to better understand and characterize the context in which the trial will take place, ultimately providing better guidance to decision-makers. These two recommendations do not themselves constitute domains of PRECIS-2-PS, but do facilitate its effective use in the design of trials of provider-focused strategies.

#### Stakeholders

Individuals involved in the trial planning phase should include representatives from research and practice to ensure that the overall intent of the trial reflects important perspectives in the decision-making process of the trial [23, 24]. Stakeholders would include (but not be limited to) members of the interdisciplinary scientific team, organizational and healthcare professional partners participating in the trial, and representatives from the settings in which the trial results are intended to be applied and adopted. All stakeholders would provide unique yet complementary input during the trial planning phase [26]. Researchers and practitioners would rely on expert and tacit knowledge about specific topics that would otherwise be inaccessible or likely inaccurate if not provided by that group. Examples include healthcare professionals identifying priority topic areas and important outcomes; statisticians providing power calculations to determine sample size; and health system leaders describing the feasibility and acceptability of the proposed provider-focused strategies. See Table 2 for more examples of the roles that stakeholders may play when planning for the trial. By involving these stakeholder groups, one would be able to address another important aspect of using PRECIS-2-PS: understanding, describing, and measuring implementation-as-usual.

**Table 2:** Stakeholder questions for designing provider-focused trials to match the overall purpose and intent of the trial

Stakeholder group	Questions
Research team	• What is the current evidence base for various provider-focused strategies? What still needs to be tested? • How many organizations (e.g., clinics, hospitals) are needed to be adequately powered to answer the primary research question? What is the likelihood that some clinics may drop out or close down and additional clinics could be recruited into the trial? • What type of RCT (e.g., cluster, stepped-wedge) is optimal for answering the research question(s)? • What analytic approach is best-equipped to answer the research question? What back-up approaches are feasible yet still rigorous if critical aspects of the trial (e.g., sample size of organizations, provider turnover) unexpectedly change during the trial?
Healthcare professionals	• What topic areas are most important to healthcare professionals within the context of service provision, scope of work, and patients’ needs? • What provider-focused strategies might be most effective and feasible given competing demands or intractable barriers that significantly constrict practice change? • What outcomes are most important to healthcare professionals within the context of their needs, patients’ needs, and professional standards? • What types of patient-focused interventions are healthcare professionals most interested in implementing or de-implementing? • What elements of the local environment (e.g., context) will be important to incorporate into trial design? • How are healthcare professionals supported in making changes to care delivery?
Information technology and/or monitoring and surveillance experts and systems	• What type of information technology systems or monitoring and surveillance models are currently available (e.g., vendor for electronic health records, digital dashboard designs)? • What changes to the information technology system or monitoring and surveillance model might be possible during and/or after the trial (e.g., order sets, shared decision-making tool, audit and feedback options)?
Quality improvement managers, technical assistance teams	• What resources are currently used to support implementation-as-usual? • Could the proposed provider-focused strategies be packaged and delivered by quality improvement managers or technical assistance teams (or other support, funding, or change management entities; e.g., technical assistance services offered by professional associations or public health agencies) during and/or after the trial? If not, who else could deliver the strategies (e.g., develop and continually update implementation toolkits, host trainings, provide external coaching)?
Healthcare delivery system leaders, public health directors	• Are the proposed provider-focused strategies feasible and acceptable? • For healthcare systems, what is the anticipated return-on-investment for delivering strategies and the downstream impact on patient outcomes relative to other potential sources of revenue, accreditation, or requirements for healthcare reimbursement? • For public health systems, can these strategies be applied to implement or de-implement additional or alternative health practices as disease topics or community health needs change over time? • If needed, would additional resources be made available to scale and sustain the provider-focused strategies after the trial?

*All stakeholders would discuss and agree on the overall purpose and intent of the trial. Subsequent discussions and decision-making for all PRECIS-2-PS domains would be consistent with the overall purpose and intent of the trial along the explanatory-pragmatic continuum

#### Implementation-as-usual

To date, most RCTs of multi-level implementation strategies (including but not limited to provider-focused strategies) use either an implementation-as-usual control condition or provide basic implementation support (e.g., 1-h training workshop, educational materials) as an enhanced implementation comparison condition. In reflecting generalizability to implementation-as-usual, it is important for trial teams to explicitly identify to what populations and settings the intent is to be generalizable, and to carefully consider the current approach to implementation in those settings. Given the importance of planning for and interpreting the results of trials, describing and defining implementation-as-usual is critically important [27, 28].

Of course, as with usual care for patients, implementation-as-usual is by no means the same across delivery sites or healthcare professionals. Improved characterization of implementation-as-usual at all trial sites at baseline and over time will offer greater understanding of to whom and where the results of the trial may be applicable. This also highlights the importance of tracking how implementation-as-usual may change over time as a result of changes to policies, reimbursement structures, or reorganization, which may enhance or limit the generalizability of study results to settings in which they are intended to be used. Note that all of these considerations similarly apply to usual care for PRECIS-2-PS, where applicable (i.e., flexibility of intervention).

## Conclusion

The PRECIS-2-PS tool for provider-focused implementation trials aims to help research and practice stakeholders plan for trials where design decisions are matched with the overall intent and purpose of the trial. The PRECIS-2-PS tool builds on PRECIS-2 by adapting all nine domains to trials testing provider-focused strategies. In doing so, it enables planning for provider-focused trials along the explanatory-pragmatic continuum, with implications for advancing the field, and ultimately maximizing the impact of trial results on improving patient outcomes, care delivery, and population health. The PRECIS-2-PS tool also responds to recent calls for a better understanding of implementation strategies. We incorporate this thinking into the PRECIS-2-PS tool by encouraging trial teams to better specify strategies [29, 30], consider adaptation of strategies [31, 32], costs of strategies [33], and describe and track changes to strategies, usual care, and implementation-as-usual over time [34] before and during the trial.

Research is needed to further develop, validate, and apply the PRECIS-2-PS tool to diverse provider-focused implementation trials. The PRECIS-2-PS tool should be formally tested for interrater reliability and discriminant validity, following the sequence of development of PRECIS-2. Applying the PRECIS-2-PS tool prospectively during trial planning, and retrospectively to published protocols, would generate an abundance of examples of domain scores, and identify aspects of the tool that may need to be refined or further specified. In addition to focusing on aspects of the tool itself, research is needed on how best to communicate trial domain scores from the PRECIS-2-PS tool. Dissemination research can help elucidate what type of information generated from the PRECIS-2-PS toolkit may be most useful for decision-makers and in what format.

Compendiums of trial case studies of PRECIS-2-PS scores would also better enable calibration of scores in the planning process and facilitate training in how to use the tool. This would help identify which PRECIS-2-PS domains are most likely to change over time and in what direction along the continuum. It would help characterize the state of provider-focused trials along the explanatory-pragmatic continuum, and identify what additional trials are needed to enhance the generalizability and applicability of strategies. It is important to remember, however, that not all provider-focused trials should be pragmatic. Explanatory trials are not inherently “bad” and pragmatic trials are not inherently “good.” Rather, each type of trial serves a purpose. Given the relatively nascent state-of-the-science of implementation strategies, one might expect to see more explanatory trials testing mechanisms of change now and more pragmatic trials testing strategies in diverse, representative settings later.

The PRECIS-2-PS tool is intended to encourage research and practice stakeholders to design provider-focused trials that match the overall intent and purpose of trials. The tool creates an opportunity to enhance research-practice partnerships by making explicit the importance and complementary role of stakeholders in the trial design phase. It also highlights the need for more and better provider-centered research [35] by focusing on priority issues, questions, and outcomes that are of interest to them. This approach is necessary for maximizing the potential of provider-focused implementation trials to have an impact once studies have been completed and papers have been published. The PRECIS-2-PS tool provides a structured approach for bringing stakeholders together in designing elements of a trial to match the intent and purpose of that trial, whether it be more explanatory, more pragmatic, or somewhere in between.

## Supplementary Information

**Additional file 1.** PRECIS-2-Provider Strategies Toolkit.

## Data Availability

Not applicable.
