# Supplementary material for: Designing provider-focused implementation trials with purpose and intent: introducing the PRECIS-2-PS tool
Source: Implement Sci. 2021 Jan 7;16:7. doi: 10.1186/s13012-020-01075-y (PMC7791810; doi:10.1186/s13012-020-01075-y)
Supplement: Supplementary file 1 — Additional file 1. PRECIS-2-Provider Strategies Toolkit. [file 13012_2020_1075_MOESM1_ESM.docx]

**PRECIS-2-Provider Strategies Toolkit**

*This toolkit provides guidance for using PRECIS-2-Provider Strategies (PRECIS-2-PS). It includes key questions to ask during the trial planning phase, a cheat sheet of the PRECIS-2-PS domains and questions, and a blank PRECIS-2-PS table to facilitate scoring for each domain during the planning phase of the trial. Information collected should be as detailed as necessary to understand and describe the contexts in which the trial will occur.*

*Note that aspects of the trial (e.g., domain scores, implementation-as-usual) may change over time due to unforeseeable circumstances or real-time necessary changes to maintain the integrity of the trial, all of which should be documented. This may include, for example, changes to recruitment procedures, primary outcome, implementation-as-usual, or policy changes (e.g., reimbursement) that may affect the trial in ways that could alter the domain to implementation-as-usual comparison or elements of the trial that can or should change along the explanatory-pragmatic continuum. This toolkit may also be used to facilitate retrospective scoring of trials, although prospective scoring is preferred to avoid recall bias and the inaccessibility or unavailability of key information not otherwise reported in trial protocols or outcome papers.*

**Key Questions**

1. **What is the overall intent and purpose of the proposed trial?**

- What are priority questions and topic areas among healthcare professionals and healthcare leadership that could be studied in a trial?
- What are the gaps in the literature and what questions still need to be answered?
- Are there particular domains that stakeholders feel should be more pragmatic or more explanatory within the trial?

1. **Who is involved in planning for and conducting the trial?** *See Table 2: Stakeholder Involvement for additional questions.*

- What unique yet complementary roles do stakeholders play as part of the trial team?
- What communication plans are in place to support engagement among the collaborative team before, during, and after the trial?
- Are there additional stakeholder groups that should be involved in the trial? If so, how can they be engaged?

1. **What is usual care and implementation-as-usual?**

- Who is best equipped to accurately describe usual care and implementation-as-usual before and during the trial?
- How often should usual care and implementation-as-usual be assessed?
- What tools are available for documenting and tracking changes to usual care and implementation-as-usual?

**PRECIS-2-PS Cheat Sheet: Domains and Key Questions**

| **Domain Name** | **Key Question** |
| --- | --- |
| 1. Eligibility | To what extent are healthcare providers in the trial similar to those in usual care? |
| 1. Recruitment | How much extra effort is made to recruit healthcare professionals into the trial compared to what is available to encourage their engagement in usual care settings? |
| 1. Setting | How different is the health care or public health setting (e.g., hospital, clinic, health department) in which the trial is conducted compared to usual care settings? |
| 1. Implementation Resources | How different are the resources needed to support the delivery of the provider-focused strategies from resources that are readily available in usual care? |
| 1. Flexibility of Provider Strategies | How different is the flexibility in how provider-focused strategies are delivered in the trial and the flexibility in how provider-focused strategies are likely to be delivered in usual care? |
| 1. Flexibility of Intervention | How different is the flexibility in how the intervention is delivered by healthcare providers to patients and the flexibility in how the intervention would be delivered in usual care? |
| 1. Data Collection | How different is the frequency and intensity of measurement and data collection throughout the trial compared to what is considered routine in usual care? |
| 1. Primary Outcome | To what extent is the trial’s primary outcome important to healthcare professionals? |
| 1. Primary Analysis | To what extent are all data included in the analysis of the primary outcome? |

**PRECIS-2-PS Trial Planning Worksheet: Domain Scores, Rationale, Usual Care and Implementation-as-Usual**

| **Domain Name** | **Score** | **Rationale** | **Description of Usual Care and Implementation-as-Usual** |
| --- | --- | --- | --- |
| 1. Eligibility |  |  |  |
| 1. Recruitment |  |  |  |
| 1. Setting |  |  |  |
| 1. Implementation Resources |  |  |  |
| 1. Flexibility of Provider Strategies |  |  |  |
| 1. Flexibility of Intervention |  |  |  |
| 1. Data Collection |  |  |  |
| 1. Primary Outcome |  |  |  |
| 1. Primary Analysis |  |  |  |

*Note*. Detailed description of usual care and implementation-as-usual is necessary for understanding and documenting the context in which the trial will occur. Stakeholders involved in trial planning are encouraged to provide as much detail as possible on the context of implementation with respect to the domains above and not be limited to a few brief descriptors. Additional trial information relevant to the score decision-making process can be added as well as changes to trial elements or the context of implementation-as-usual that may occur during the trial.
